# Marked Asymptomatic Creatine Kinase Elevation With Preserved Renal Function Identified Through Protocol-Driven Monitoring in a Clinical Trial Participant: A Case Report

**DOI:** 10.7759/cureus.112027

**Published:** 2026-07-03

**Authors:** Raymond T Anasobi, Taranjeet Parmar, Omar Stewart

**Affiliations:** 1 Internal Medicine, Healthcare Research Network, Tinley Park, USA; 2 Family Medicine, Saint James School of Medicine, Park Ridge, USA; 3 Internal Medicine/Orthopedic Surgery, All Saints University School of Medicine, Roseau, DMA

**Keywords:** adverse event monitoring, asymptomatic hyperckemia, clinical trial participation, clinical trial safety, creatine kinase elevation, creatine phosphokinase, metabolic abnormalities, preserved renal function, protocol-driven monitoring, rhabdomyolysis

## Abstract

Elevated creatine kinase (CK) levels may occur in a wide range of clinical conditions including skeletal muscle injury, inflammatory myopathies, medication-associated myotoxicity, rhabdomyolysis, and strenuous physical activity. Distinguishing clinically significant muscle injury from transient physiologic hyperCKemia may be particularly challenging in asymptomatic patients undergoing routine laboratory surveillance. We report the case of a 62-year-old African American male clinical trial participant in whom marked asymptomatic hyperCKemia was identified through protocol-driven safety monitoring during long-term study participation. Serial laboratory assessments identified a marked isolated serum CK elevation (2694 U/L) with preserved creatinine clearance and stable renal function throughout the monitoring period. Approximately three weeks prior to the peak laboratory abnormality, the participant reported intensification of physical exercise activity. Despite marked CK elevation, the participant denied myalgia, muscle weakness, dark urine, decreased urine output, chest pain, palpitations, dyspnea, or constitutional symptoms. Twelve-lead electrocardiography demonstrated normal sinus rhythm with nonspecific low-amplitude T-wave abnormalities without clinically significant arrhythmias or acute ischemic abnormalities. Review of temporal relationships, concomitant medications, available safety data, and the Investigator’s Brochure did not support the investigational product as a likely contributor to the laboratory abnormalities. The participant remained clinically stable with preserved renal function and spontaneous improvement in laboratory abnormalities during continued monitoring. This case highlights the importance of contextual interpretation of marked asymptomatic CK elevation and demonstrates how protocol-driven safety monitoring can facilitate early identification and evaluation of clinically silent laboratory abnormalities in clinical trial participants.

## Introduction

Creatine kinase (CK) is an intracellular enzyme released following skeletal muscle injury, myocardial injury, or increased muscle membrane permeability [1]. Elevated serum CK levels may occur in a broad spectrum of clinical conditions including inflammatory myopathies, medication-associated myotoxicity, metabolic disorders, trauma, rhabdomyolysis, and strenuous exercise [2,3]. Although marked CK elevations frequently raise concern for clinically significant muscle injury and potential renal complications, asymptomatic hyperCKemia remains diagnostically challenging, particularly when identified incidentally during routine laboratory monitoring in otherwise clinically stable individuals. 

Exercise-associated hyperCKemia is increasingly recognized among physically active individuals participating in high-intensity resistance or endurance exercise [4]. In some cases, substantial laboratory abnormalities may occur despite absence of overt symptoms, complicating differentiation between physiologic skeletal muscle adaptation and evolving rhabdomyolysis [5]. Comprehensive evaluation incorporating renal function assessment, symptom review, medication review, and electrocardiographic findings is therefore essential in contextualizing the clinical significance of elevated CK levels.

Structured protocol-driven laboratory monitoring within clinical trial settings provides a unique opportunity for early identification of clinically silent physiologic and metabolic abnormalities that may otherwise remain undetected during routine outpatient care. Similar longitudinal monitoring strategies have previously demonstrated utility in identifying asymptomatic metabolic deterioration during clinical trial participation [6]. However, reports describing marked asymptomatic hyperCKemia with preserved renal function identified through longitudinal clinical trial monitoring remain relatively uncommon. 

We report a case of marked asymptomatic hyperCKemia identified through protocol-driven safety monitoring in a clinical trial participant with preserved renal function and stable electrocardiographic findings. This case highlights the importance of cautious clinical interpretation and comprehensive safety assessment when evaluating isolated laboratory abnormalities in otherwise clinically stable patients.

## Case presentation

A 62-year-old African American male participating in a clinical trial was found to have marked asymptomatic hyperCKemia during routine protocol-mandated safety monitoring. The participant's medical history was significant for hypertension, gout, type 2 diabetes mellitus, diabetic peripheral neuropathy, and hypercholesterolemia.

The participant had initially completed approximately four months of randomized double-blind study participation before transitioning into a 52-week open-label extension evaluating an investigational therapy targeting peripheral pain signaling pathways in diabetic peripheral neuropathy. Body weight was 89 kg with a body mass index of 27.3 kg/m².

Concomitant medications included amlodipine 5 mg daily, colchicine 0.6 mg daily, hydrochlorothiazide 12.5 mg daily, metoprolol 50 mg daily, and atorvastatin 40 mg daily. No recent medication additions, dosage adjustments, or treatment interruptions were reported prior to the identification of the CK elevation.

**Figure 1:**
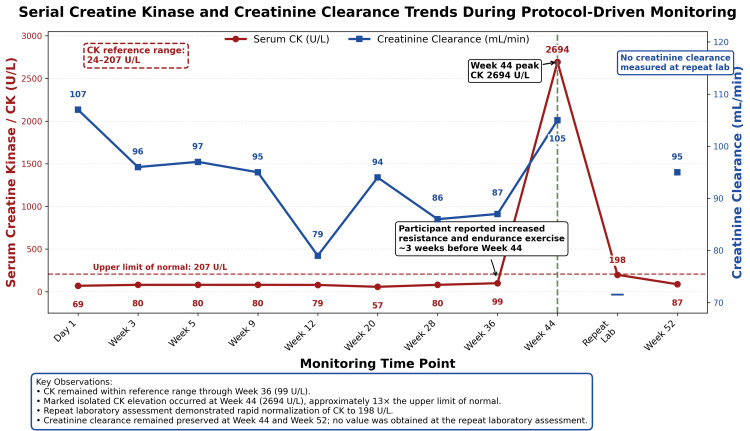
Serial CK and Creatinine Clearance Trends During Protocol-Driven Monitoring Serial laboratory monitoring demonstrated a marked isolated elevation in serum CK at Week 44 (2694 U/L; approximately 13x the upper limit of normal) with preserved creatinine clearance throughout follow-up. Repeat Lab refers to an unscheduled confirmatory laboratory assessment performed approximately one week after the Week 44 visit to evaluate the elevated CK level. Creatinine clearance was not measured during the repeat assessment. CK: creatine kinase

Serial laboratory assessments demonstrated marked isolated elevation in serum CK levels during longitudinal monitoring while creatinine clearance remained preserved throughout study participation. Baseline and serial renal indices remained stable throughout follow-up without evidence of clinically significant renal impairment. These findings are summarized in Figure 1 and Tables 1, 2.

**Table 1:** Serial Creatine Kinase and Creatinine Clearance Trends During Protocol-Driven Monitoring CK: creatine kinase. Creatinine clearance was not measured during the repeat laboratory assessment performed after the Week 44 peak CK elevation. CK reference range: 24 - 207 U/L

Time Point	CK (U/L)	Creatinine Clearance (ml/min)
Day 1	69	107
Week 3	80	96
Week 5	80	97
Week 9	80	95
Week 12	79	79
Week 20	57	94
Week 28	80	86
Week 36	99	87
Week 44	2694	105
Repeat Lab	198	-
Week 52	87	95

**Table 2:** Laboratory and Urinalysis Findings During Peak HyperCKemia at Week 44 CK: creatine kinase; AST: aspartate aminotransferase; ALT: alanine aminotransferase; BUN: blood urea nitrogen. Laboratory values were obtained at the Week 44 study visit during peak CK elevation (2694 U/L). Renal function remained preserved, with normal serum creatinine and clearance. Urinalysis demonstrated no clinically significant abnormalities, including no evidence of hematuria, proteinuria, glucosuria or ketonuria. A repeat laboratory assessment performed approximately one week later demonstrated normalization of CK levels (198 U/L).

Parameter	Result/Units	Reference Range
CK	2694 U/L	24 - 207
AST	42 U/L	10 - 43
ALT	31 U/L	10 - 40
Alkaline Phosphate	69 U/L	43 - 115
Bilirubin, Total	0.43 mg/dl	<=1.1
Bilirubin Direct	0.20 mg/dL	<= 0.40
Protein	7.6 g/dL	6.0 - 8.0
Creatinine	0.9 mg/dL	0.6 - 1.2
BUN	17 mg/dL	5 - 20
Creatinine Clearance (Estimated by Weight)	105 ml/min	85 - 125
Sodium	145 mEq/L	133 - 145
Potassium	5.0 mEq/L	3.5 - 5.0
Bicarbonate	21 mEq/L	21 - 33
Chloride	107 mEq/L	95 - 110
Calcium	10.1 mg/dL	8.5 - 10.5
Magnesium	1.8 mEq/L	1.3 - 2.1
Phosphorus	3.2 mg/dL	2.5 - 4.5
Urinalysis		
Glucose, Urine	Negative mg/dL	Negative
Protein	Negative mg/dL	Negative
Bilirubin	Negative	Negative
Urobilinogen	0.2 mg/dL	0.2, 1.0
pH	5.0	5.0, 6.0, 7.0, 8.0
Blood	Negative	Negative
Ketone	Negative mg/dL	Negative
Specific Gravity	1.019	1.002 - 1.035

Approximately three weeks prior to the Week 44 study visit, the participant specifically reported increasing the intensity and frequency of resistance and endurance exercise compared with his usual routine. The temporal relationship between escalation in exercise intensity and marked CK elevation raised the possibility of exercise-associated hyperCKemia as a plausible contributing factor. However, causality could not be definitively established.

Despite marked CK elevation, the participant denied myalgia, muscle weakness, muscle cramping, dark urine, decreased urine output, fever, fatigue, chest pain, dyspnea, palpitations, dizziness, alcohol use, recent trauma, falls, seizures, recent intramuscular injections or constitutional symptoms. Physical examination remained unremarkable throughout the monitoring period. Vital signs remained stable without evidence of systemic illness or volume depletion.

Renal function remained preserved throughout follow-up despite the marked CK elevation. Serum creatinine, creatinine clearance, electrolyte, hepatic indices, and urinalysis findings remained within reference ranges, with no evidence of metabolic acidosis or acute kidney injury (Table 2). These findings argue against clinically significant rhabdomyolysis-associated renal injury despite the marked transient hyperCKemia [7]. 

Given the participant’s involvement in a clinical trial, a comprehensive assessment of the investigational product was performed. Review of temporal relationships, available safety data, concomitant medications, and the Investigator's Brochure did not identify elevated creatine kinase levels or rhabdomyolysis as known or expected adverse effects associated with the investigational product. While no evidence directly implicated the investigational product, a causal relationship could not be definitively excluded.

**Figure 2:**
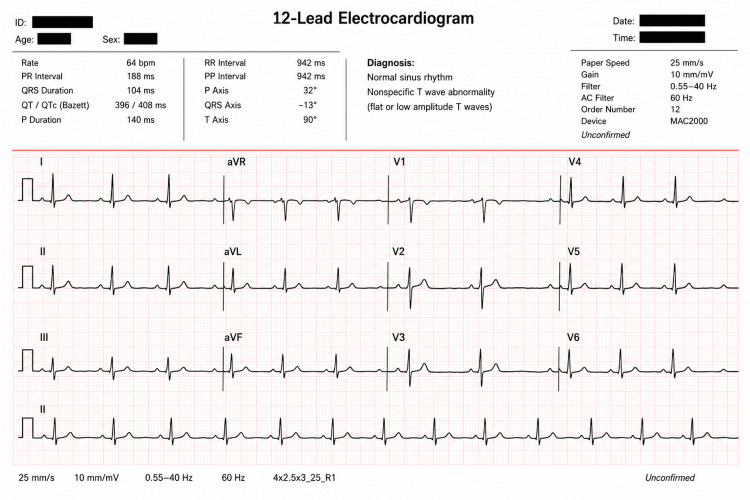
Twelve-Lead Electrocardiogram Demonstrating Normal Sinus Rhythm and Nonspecific T-Wave Abnormalities During Peak CK Elevation Twelve-lead electrocardiogram obtained during peak CK elevation (2694 U/L) demonstrated normal sinus rhythm with nonspecific low-amplitude T-wave abnormalities without clinically significant arrhythmias, conduction abnormalities, or acute ischemic changes. Patient identifiers were redacted to preserve confidentiality. CK: creatine kinase

Electrocardiographic evaluation was obtained during assessment of the peak laboratory abnormality. Twelve-lead electrocardiography demonstrated normal sinus rhythm with nonspecific low-amplitude T-wave abnormalities and no clinically significant arrhythmias, acute ischemic changes, or conduction abnormalities (Figure 2).

The participant had remained on stable doses of atorvastatin and colchicine without recent dose adjustments, treatment interruptions or introduction of interacting therapies before the observed laboratory abnormality. Although both medications have recognized myopathic potential, their long-term stable use without recent changes made an acute medication-related effect less certain.

The participant was counseled regarding exercise intensity modification, hydration optimization, and continued longitudinal monitoring. Follow-up assessments demonstrated ongoing clinical stability with preserved renal function and spontaneous improvement in laboratory abnormalities without evidence of systemic complications.

## Discussion

This case highlights several clinically important aspects of asymptomatic hyperCKemia identified through structured protocol-driven laboratory monitoring. Although elevated serum CK levels frequently raise concern for rhabdomyolysis, inflammatory myopathy, medication-associated myotoxicity, or severe systemic muscle injury, substantial laboratory abnormalities may occur in otherwise clinically stable individuals [2,4].

Approximately three weeks prior to the peak laboratory abnormality, the participant reported significantly intensifying physical exercise activity. The temporal association between increased exercise intensity and significant CK elevation supports exercise-associated skeletal muscle enzyme release as a plausible contributing factor. However, definitive causality cannot be established given the observational nature of this report and absence of objective exercise quantification or muscle-specific diagnostic testing.

Importantly, the participant remained asymptomatic throughout the monitoring period with preserved renal function, stable creatinine clearance, and reassuring electrocardiographic findings. Acute kidney injury represents one of the most clinically significant complications of rhabdomyolysis and is frequently mediated through myoglobin-associated renal tubular injury, oxidative stress, and volume depletion [7]. Continued renal stability argued against clinically significant rhabdomyolysis-associated renal injury despite substantial laboratory abnormalities.

This case additionally demonstrates the importance of integrating objective laboratory trends with clinical assessment during safety monitoring in clinical trial participants. Protocol-driven assessments enabled early identification of clinically silent laboratory abnormalities that may otherwise have remained undetected in routine outpatient care. Comprehensive evaluation including exercise history, symptom assessment, renal indices, medication review, electrocardiographic findings, and investigational product safety data allowed for contextual interpretation of the observed abnormalities.

Electrocardiographic assessment further strengthened the overall safety evaluation. Significant muscle injury and rhabdomyolysis may be associated with electrolyte disturbances capable of precipitating clinically important arrhythmias or conduction abnormalities [8]. The absence of clinically significant electrocardiographic instability supported the participant’s overall physiologic stability and reduced concern for severe systemic complications.

Available safety information, temporal relationships, concomitant medications, and review of the Investigator's Brochure did not identify evidence strongly supporting investigational product involvement. However, given the observational nature of this report, a causal relationship could not be definitively excluded.

This report also highlights the growing importance of contextual interpretation of laboratory abnormalities among physically active individuals. Increasing participation in resistance and endurance exercise programs may contribute to asymptomatic elevations in CK and other laboratory abnormalities that mimic clinically significant pathology [5,9]. Careful interpretation of laboratory trends within the context of exercise history and overall clinical stability therefore remains essential to avoid unnecessary diagnostic escalation or inappropriate discontinuation of investigational therapies.

Recent literature has additionally demonstrated that asymptomatic or minimally symptomatic hyperCKemia may occur in clinically stable individuals without progression to clinically significant rhabdomyolysis or renal compromise [10,11]. Serial renal monitoring and clinical assessment therefore remain essential in differentiating benign physiologic laboratory abnormalities from evolving systemic pathology.

Furthermore, concomitant medications including statins and colchicine have independently been associated with skeletal muscle enzyme elevation and myotoxicity [12,13]. Although the participant had tolerated both medications for an extended period without recent dose modification, the known myopathic potential of concomitant statin and colchicine therapy represents an additional plausible contributing factor to the observed CK elevation. However, no definitive evidence of medication-associated toxicity was identified in this participant, and the presence of concomitant therapies with known myopathic potential further supported the need for continued structured monitoring and comprehensive clinical assessment. 

Additional causes of asymptomatic CK elevation, including inflammatory myopathies, hypothyroidism, viral myositis, occult trauma, seizures, alcohol-related muscle injury, and electrolyte disturbances, should also be considered when evaluating marked hyperCKemia. Although comprehensive testing for all potential etiologies was not performed in this case, the absence of progressive symptoms, preserved renal function, stable electrocardiographic findings, and spontaneous normalization of CK levels reduced concerns for an evolving systemic myopathic process. 

Several limitations should be acknowledged. Quantitative exercise metrics, CK-MB fractionation, cardiac troponin testing, repeat electrocardiographic assessments, serum myoglobin concentrations, and advanced neuromuscular testing were not available. Consequently, definitive attribution of the observed laboratory abnormalities to exercise, medication effects, or other subclinical etiologies could not be established. Nevertheless, the longitudinal nature of protocol-driven monitoring provided detailed characterization of laboratory trends and clinical outcomes over time. The longitudinal assessment strengthened interpretation of the observed CK elevation by providing pre-event, peak-event, and follow-up data points unavailable in many routine clinical encounters. 

## Conclusions

This case illustrates how protocol-driven longitudinal monitoring can identify marked asymptomatic hyperCKemia in otherwise clinically stable individuals with preserved renal function and clinical stability.

Comprehensive evaluation incorporating serial laboratory trends, renal indices, electrocardiographic findings, medication review, symptom assessment, and available safety information is essential when evaluating isolated laboratory abnormalities in clinical trial participants. Although multiple potential contributing factors were identified, definitive causality could not be established based on the available clinical and laboratory data. This report highlights the value of structured protocol-driven monitoring in contextualizing clinically silent laboratory abnormalities and guiding appropriate clinical decision-making. 
